# Comparative efficacy of intravascular ultrasound and fractional flow reserve in guiding percutaneous coronary intervention

**DOI:** 10.1097/MD.0000000000041743

**Published:** 2025-03-21

**Authors:** Huiting Wu, Xingan Wu, Wen Yu, Han Wang, Baozhen Tan, Liang Hou, Jilin Xu

**Affiliations:** a Department of Cardiology, General Hospital of The Yangtze River Shipping, Wuhan Brain Hospital, Wuhan, China.

**Keywords:** fractional flow reserve, intermediate coronary lesions, intravascular ultrasound, percutaneous coronary intervention

## Abstract

This study aimed to compare the postoperative function of patients with critical coronary artery lesions undergoing intervention guided by intravascular ultrasound (IVUS) vs those guided by fractional flow reserve (FFR). A total of 226 patients (293 lesions) with coronary angiography-confirmed stenosis of 40% to 70% were enrolled and divided into 3 groups: the IVUS-guided group (98 lesions), the FFR-guided group (101 lesions), and the medical treatment group (94 lesions). In the IVUS-guided group, coronary stent implantation was performed if the minimum lumen area at the stenosis was < 4 mm^2^. In the FFR-guided group, intervention was performed if FFR < 0.8. Patients were followed for 1-year postoperatively, and the incidence of major adverse cardiovascular events (MACE), including death, myocardial infarction, and target vessel revascularization, was compared among the 3 groups. There were no significant differences in the degree of stenosis or lesion length among the 3 groups as determined by coronary angiography. The proportion of patients undergoing coronary intervention was significantly higher in the IVUS-guided group compared to the FFR-guided group (*P* < .001). However, there was no significant difference in the incidence of MACE among the 3 groups (*P* = .182). This study found no significant difference in MACE between the 3 guidance strategies – IVUS, FFR, and angiography – in patients with intermediate coronary lesions undergoing PCI. These findings suggest that, in this patient population, the choice of guidance method may not impact MACE outcome.

## 1. Introduction

Intermediate coronary lesions refer to lesions observed during coronary angiography that are moderately narrowed, typically ranging from 40% to 70%. Currently, there is no unified standard for defining moderate stenosis. Some literature suggests a degree between 50% and 70%, while others define it as stenosis ranging from 40% to 80%. Intermediate coronary lesions are common in coronary angiography and interventions. Studies have shown that 6% of borderline lesions progress to acute coronary events requiring intervention within a year. Additionally, 87% of lesions requiring intervention were previously classified as borderline lesions with <60% stenosis on angiography.^[1]^ The PROSPECT study also revealed that 11% of coronary events arise from non-culprit vessels (vessels not originally responsible for symptoms) with borderline lesions.^[2]^ The impact of intervention on the prognosis of all borderline lesions remains uncertain. Latacz et al’ s study highlights that pharmacological treatment of intermediate coronary lesions results in fewer adverse events such as restenosis and recurrent angina compared to angiography-guided percutaneous coronary intervention (PCI), emphasizing the need for functional assessment tools such as IVUS or FFR to guide interventions.^[3]^ Although coronary angiography serves as the gold standard for coronary anatomical evaluation, it has several limitations, particularly in assessing moderate stenosis, as it provides only 2-dimensional images of contrast agent filling from certain angles, and the assessment of stenosis severity relies on comparison with neighboring “normal vessels.”^[4–7]^ Therefore, we need to further examine and select patients with relatively high-risk borderline lesions for intervention. Intravascular ultrasound (IVUS) can provide real-time cross-sectional images of coronary arteries, which can more accurately assess the degree of stenosis compared to coronary angiography. Studies have shown that for coronary arteries with a diameter > 2.5 mm (excluding the left main coronary artery), a minimal lumen area (MLA) < 4 mm^2^ in the proximal and mid segments may lead to myocardial ischemia. Delaying coronary intervention for lesions with MLA > 4 mm^2^ may lead to beneficial clinical outcomes.^[8–10]^

Coronary angiography and intravascular ultrasound examinations indirectly infer the presence of myocardial ischemia by assessing the degree of coronary stenosis. However, fractional flow reserve (FFR) measurement allows us to directly evaluate the physiological significance of borderline lesions functionally and is currently considered the gold standard for assessing myocardial ischemia. Multiple studies have shown that in cases with FFR > 0.80, delaying PCI treatment and adopting standard medical therapy for coronary artery disease is safe and reliable. Conversely, performing PCI in patients with FFR < 0.80 can reduce the occurrence of some acute ischemic events.^[11–15]^ Currently, research remains limited on determining the most suitable method for evaluating borderline lesions, with no global consensus on the optimal approach.

This study aims to fill that gap by investigating 586 coronary lesions (40%–70% stenosis) verified through angiography. These lesions were divided into study and control groups. In the study group, patients underwent further evaluation using either IVUS or FFR to guide their treatment strategy, while the control group received standard medical therapy. By tracking the occurrence of major adverse cardiovascular events (MACE) over the course of 1 year, this study seeks to clarify which diagnostic and treatment approach most effectively improves patient outcomes, providing valuable insights into the clinical management of borderline coronary lesions.

## 2. Materials and methods

### 2.1. Study population

This study was approved by the Ethics Committee of the General Hospital of the Yangtze River Shipping. From January 2010 to March 2012, we meticulously selected inpatients who underwent coronary angiography at our hospital. These patients had proximal and mid-segment coronary lesions with diameters exceeding 2.5 mm, and the stenosis degree ranged from 40% to 70%. Additionally, they either failed to have myocardial ischemia confirmed through noninvasive tests, had inconclusive test results, or could not undergo noninvasive myocardial ischemia testing for various reasons. Exclusion criteria were as follows: patients undergoing emergency PCI due to acute coronary syndrome; patients with previous coronary artery bypass graft surgery; patients with multiple lesions in the same artery; patients with left main disease, cardiomyopathy, or other life-threatening conditions; patients with contraindications to adenosine, aspirin, or clopidogrel. Patients were sequentially numbered according to the time of surgery and randomly allocated to the IVUS group, FFR group, or defer group using a random number table. In the IVUS group, if MLA < 4 mm^2^, coronary stent implantation was performed; in the FFR group, an FFR < 0.80 was used as an indication for coronary stent implantation; while the defer group received only standard medication for coronary artery disease.

### 2.2. Research methods

#### 2.2.1. Coronary angiography

During the coronary angiography of the left and right coronary arteries, the Judkins technique was employed, administering 200 µg of nitroglycerin intracoronarily to each patient. This was done to effectively eliminate the possibility of coronary artery spasm. A 6F angiographic catheter was used to ensure data accuracy. The DCI software of the angiography machine was utilized to measure the degree and length of stenosis of the target lesions, providing data support for subsequent treatment.

#### 2.2.2. Intravascular ultrasound measurement

In diagnosing and guiding the treatment of coronary artery disease, we utilized the iLab Ultrasound Imaging System with the Atlantis SR Pro coronary ultrasound catheter. This 2.9 F catheter, featuring advanced IVUS technology, allows for precise imaging and is ideal for navigating narrow vessels. With a 40 MHz frequency, it provides high-resolution images, enabling detailed assessment of lesions. The catheter was advanced to the distal lesion using a 0.014-inch guidewire, and images were acquired during automated pullback at 0.5 mm/s. Measurements such as minimum lumen area (MLA), lesion length, plaque burden, and stent deployment were taken following American College of Cardiology guidelines. Analysis was performed by a blinded reviewer using CVIS ClearView or Boston Scientific iReview software, with calibrations based on internal markers. All measurements were done at end-diastole at the narrowest point of the vessel.

#### 2.2.3. FFR measurement

FFR was measured using the RadiAnalyzer Xpress device manufactured by St. Jude Medical. The PressureWireTM Certus pressure wire was placed at least 3 cm distal to the target lesion to measure intracoronary pressure, while a 6F guiding catheter was used to measure pressure at the aortic root or coronary ostium. After obtaining the resting Pd/Pa value, adenosine (140 μg·kg^-1^·min-1, 2–3 minutes) was intravenously infused via the brachial vein to induce maximal hyperemia, and the FFR value was recorded at the time of maximum pressure gradient.

#### 2.2.4. Clinical follow-up

All enrolled patients received clinical follow-up at 1, 3, 6, and 12 months after enrollment (with follow-up personnel blinded to patients’ coronary angiography results and group allocation). Medication use and adverse events (including myocardial infarction, revascularization of target lesions due to ischemia, and death) were recorded. Diagnosis of myocardial infarction followed the description of spontaneous myocardial infarction in the 2012 Global Myocardial Infarction Unified Definition. Revascularization of target lesions included both stent implantation and coronary artery bypass grafting. Death encompassed all deaths occurring for any reason during the follow-up period.

### 2.3. Statistical analysis

**Figure 1.**
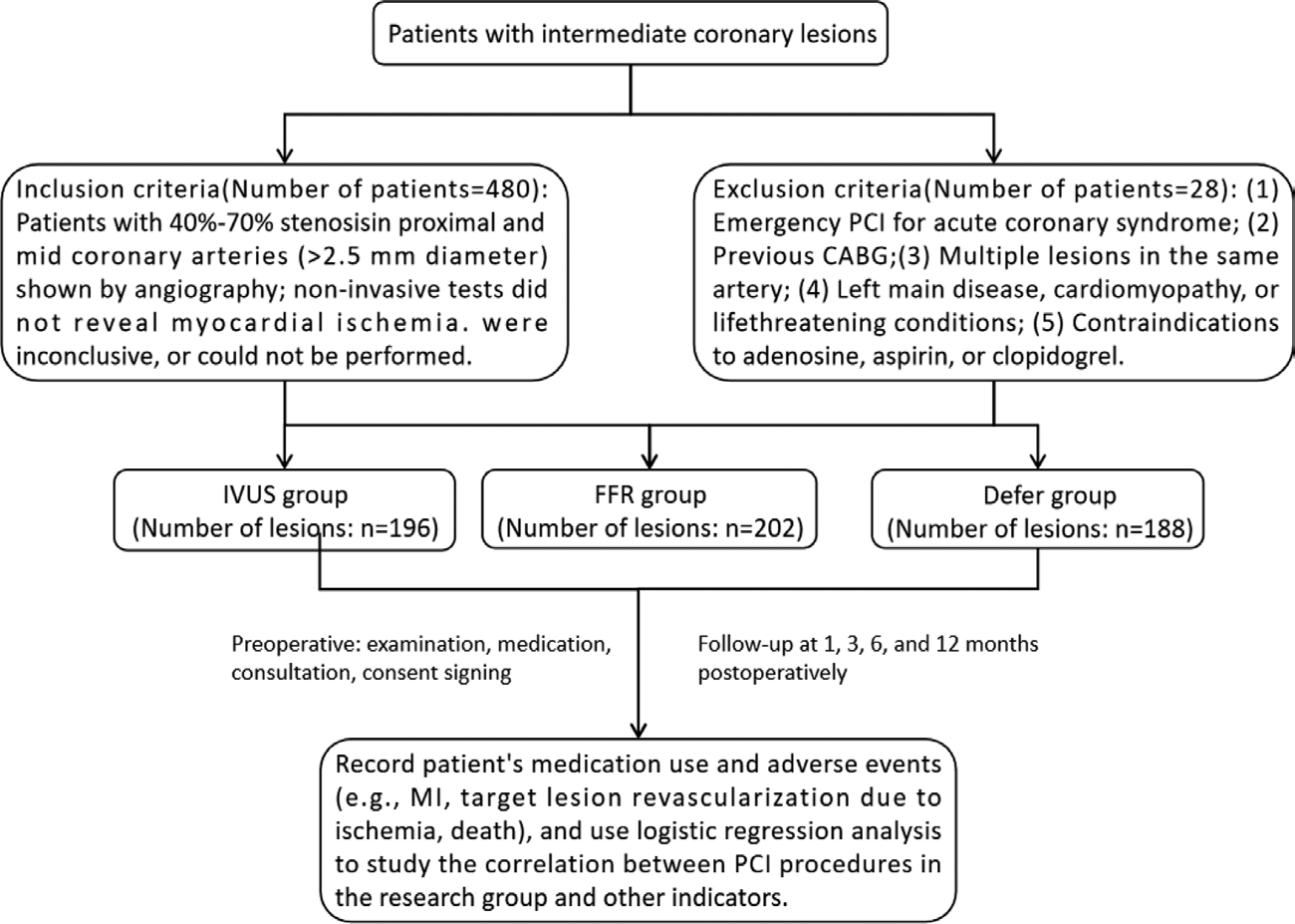
Research flowchart.

Statistical analysis was performed using SPSS 15.0 software package (Chicago). Continuous data were presented as mean ± standard deviation. Independent sample t-test was used for comparison of means between 2 groups, while chi-square test and Fisher exact test were employed for comparison of frequency data between groups. A significance level of *P* < .05 was considered statistically significant. Logistic regression analysis was utilized to explore the correlation between PCI procedures performed in the research group and other indicators. The flowchart of this study is illustrated in Figure 1.

## 3. Results

We initially selected 480 patients as the research sample. However, during the continuous clinical follow-up, 28 patients were unable to complete the follow-up due to various reasons, which somewhat affected the completeness of our data. Despite this, 452 patients were still included in the study, among whom a total of 586 intermediate lesions were identified. To better present the clinical characteristics of these 452 patients, their general clinical information is organized in Table 1.

**Table 1:** Baseline clinical and lesion-specific characteristics of the patients.

Clinical	IVUS group (n = 196)	FFR group (n = 202)	Defer group (n = 188)	*P*
Age	67 ± 18	62 ± 17	63 ± 15	.012
Men	110	124	112	.746
Diabetes	54	60	72	.240
Hypertension	108	104	104	.831
Hypercholesterolemia	118	122	110	.958
Current smoking	118	108	120	.326
Previous PCI	58	78	46	.095
Stable angina	150	158	132	.400
ACS	46	44	56	.400
LVEF (%) angiography	55 ± 6	57 ± 7	54 ± 8	.137
Multi	110	112	90	.447
LAD	88	104	82	.493
Proximal	94	106	88	.702
Lesion length	22 ± 13	25 ± 12	23 ± 12	.126
Percent diameter stenosis (%)	53 ± 8	55 ± 10	52 ± 8	.185

ACS = acute coronary syndrome, LAD = left anterior descending, LVEF = left ventricular ejection fraction.

Upon detailed analysis of the baseline characteristics of the 3 patient groups, we found no significant differences in age, gender, and distribution of coronary heart disease risk factors. Similarly, meticulous observation through coronary angiography revealed no notable differences in the degree of coronary stenosis, lesion length, and specific lesion locations among the 3 groups, whether in single-vessel or multi-vessel disease distribution. However, further data analysis showed that 94 lesions in the IVUS group underwent PCI (48%), while in the FFR group, 46 lesions received PCI (22.8%). This comparison clearly indicates that the proportion of interventional treatment in the IVUS group was significantly higher than in the FFR group (*P* < .001, see Table 2).This finding highlights a significant trend toward higher intervention rates in the IVUS group, suggesting differences in the treatment approach between IVUS-guided and FFR-guided strategies.

**Table 2:** Procedural results in IVUS and FFR groups.

	IVUS group		FFR group		*P*
	Defer (n = 204)	PCI (n = 188)	Defer (n = 312)	PCI (n = 92)	
IVUS pre-interventional MLA (mm^2^)	3.60 ± 0.67	3.12 ± 0.49			
Plaque burden (%)	63.2 ± 8.5	70.3 ± 7.4			<.01*
Post-intervention MSA (mm^2^)		7.21 ± 0.57			
FFR Pre-interventional			0.88 ± 0.07	0.71 ± 0.06	
Post-interventional				0.93 ± 0.05	
Stent number		1.2 ± 0.4		1.1 ± 0.3	.214
Stent length (mm)		24 ± 9.3		22 ± 8.6	.374
Stent size (mm)		2.92 ± 0.33		2.90 ± 0.30	.835

FFR = fractional flow reserve, IVUS = intravascular ultrasound, MLA = minimal lumen area, MSA = minimum stent area, PCI = percutaneous coronary intervention.

*Indicates statistically significant.

When performing logistic regression analysis on the data from the FFR group, the results indicated that an FFR value below 0.80 was significantly associated with lesions located in the left anterior descending artery (LAD) and with the length of the lesion. Specifically, when the FFR value was below 0.80, the risk of the lesion appearing in the LAD was 2.52 times higher than in other locations (OR = 2.52, *P* = .004). Additionally, the risk associated with increased lesion length was also significant, with a 3.62 times higher risk (OR = 3.62, *P* = .003). These detailed data are recorded in Table 3, providing profound insights into the relationship between FFR values, lesion location, and lesion length. This analysis underscores the importance of considering both the FFR value and the specific characteristics of the lesions, such as their location in the LAD and their length, when determining the appropriate treatment strategy for coronary intermediate lesions.

**Table 3:** Multivariable logistic regression of the correlation of a FFR < 0.8 with subsequent PCI.

Variable	Odds ratio	95% confidence interval	*P*
Diameter stenosis (%)	1.07	1.03 to 1.12	.035
Lesion length	3.62	1.64 to 6.83	.003
Multi (vs single) VD	1.01	0.93 to 2.35	.224
LAD lesions (vs RCA)	2.52	1.25 to 5.67	.004
LCX lesions (vs RCA)	0.83	0.56 to 1.28	.186

FFR = fractional flow reserve, LCX = left circumflex artery, PCI = percutaneous coronary intervention, RCA = right coronary artery, VD = vascular disease.

In the FFR group, logistic regression analysis revealed a significant association between FFR < 0.80 and lesions located in the left anterior descending artery (OR = 2.52, *P* = .004) as well as lesion length (OR = 3.62, *P* = .003, Table 3).

In the IVUS group, logistic regression analysis demonstrated a strong correlation between undergoing PCI procedures and plaque burden (OR = 3.21, *P* = .003, Table 4).

**Table 4:** Multivariable logistic regression of the correlation of a MLA < 4 mm^2^ with subsequent PCI.

Variable	Odds ratio	95% confidence interval	*P*
Diameter stenosis (%)	1.87	1.24 to 3.24	.008
Lesion length	0.82	0.64 to 1.83	.033
Multi (vs single) VD	1.25	0.89 to 2.86	.304
LAD lesions (vs RCA)	1.49	1.09 to 3.71	.011
LCX lesions (vs RCA)	0.79	0.25 to 1.11	.215
Plaque burden	3.21	1.05 to 6.49	.003

LCX = left circumflex artery, MLA = minimal lumen area, PCI = percutaneous coronary intervention, RCA = right coronary artery, VD = vascular disease.

**Figure 2.**
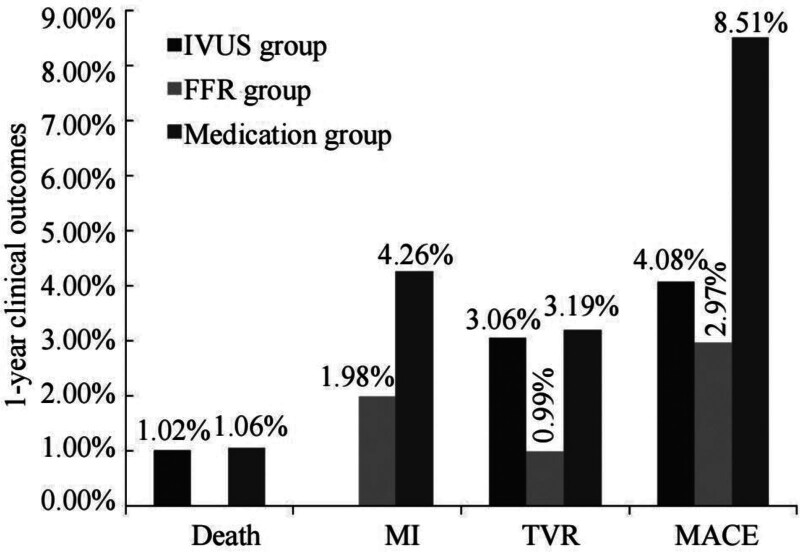
Adverse clinical events in the 3 groups at 1 yr following the procedure. MACE = major adverse cardiac event (*P* > .05), MI = myocardial infarction, TVR = target vessel revascularization.

During the follow-up period, in the IVUS group, the incidence of MACE was 4%. This included 1 patient who died due to major bleeding post-PCI, 1 patient who underwent target vessel revascularization due to in-stent restenosis, and the remaining 2 patients who underwent target vessel revascularization were in the defer group. In the FFR group, the incidence of MACE was 3%, all occurring in patients receiving medical treatment. In the defer group, the incidence of MACE was 8.5%, with 1 patient dying due to extensive cerebral infarction. There was no statistically significant difference in the incidence of MACE among the 3 groups (*P* = .182, Figure 2).

## 4. Discussion

IVUS offers several advantages over coronary angiography, including more accurate assessment of coronary stenosis and the ability to analyze plaque characteristics, select stent size, and evaluate stent apposition post-intervention. Chang-Wook et al classified intermediate coronary lesions into 2 groups based on IVUS-derived MLA < 4 mm^2^ as the indication for intervention in 1 group and FFR < 0.8 as the indication for intervention in the other group. The study found a significantly higher rate of intervention in the IVUS group compared to the FFR group (91.5% vs 33.7%, *P* < .001), with no significant difference in the incidence of MACE between the 2 groups at 1-year follow-up.^[16,17]^ However, this study did not include a control group, and did not compare the incidence of cardiovascular adverse events between these 2 examination methods and solely medical treatment or intervention treatment strategy based on coronary angiography. Therefore, in this study, 240 patients were enrolled and divided into the IVUS group, FFR group, and defer group. After 1 year of follow-up, the results showed a significantly higher rate of intervention in the IVUS group compared to the FFR group, with no difference in the incidence of MACE between the 2 groups, consistent with previous research findings. The higher rate of intervention in the IVUS group may be related to its single MLA criterion. Coronary arteries vary in diameter from proximal to distal, and the impact of lesions located at the proximal or distal ends of the coronary artery is evidently different. Koo et al^[18]^ evaluated the optimal diagnostic criteria and diagnostic accuracy of IVUS for functionally significant stenotic lesions (FFR < 0.8) in 267 patients with borderline lesions. The results showed that factors determining FFR included MLA and lesion location. Using FFR < 0.8 as the predictive threshold, the optimal MLA threshold for the proximal segment of the left anterior descending artery was 3.0 mm^2^, and for the mid-segment of the left anterior descending artery before the origin of the second diagonal branch was 2.75 mm^2^. Therefore, it may be inappropriate to evaluate all lesions based on a single MLA value. Is setting different MLA thresholds based on vessel diameter able to improve these results? Currently, there are no related studies reported. Ben-Dor et al^[19]^ studied 92 borderline lesions in 84 patients with vessel diameters > 2.5 mm and found that for vessel reference diameters of 2.5 to 3.0 mm, 3.0 to 3.5 mm, and > 3.5 mm, the optimal MLA thresholds for predicting FFR < 0.75 were 2.6 mm^2^, 2.8 mm^2^, and 3.7 mm^2^, respectively. Therefore, IVUS can be used to guide the intervention strategy for borderline lesions, but relying solely on a single MLA value as the standard for evaluating all lesions is evidently insufficient.

Studies on fluid dynamics have shown that the decrease in pressure gradient after fluid passes through a constriction in a conduit depends on 3 main parameters: the minimum cross-sectional area of the constriction, the length of the constriction, and the velocity of the fluid. FFR is considered to encompass these 3 aspects, thus allowing for a comprehensive evaluation of the functional significance of a stenotic lesion. However, the current standards for intravascular ultrasound (IVUS) only evaluate the functional significance of lesions based on 1 aspect, namely, the measurement of MLA. This approach is evidently insufficient, as recent studies have highlighted that MLA alone may not adequately determine the need for PCI. Specifically, MLA values can vary significantly depending on the vessel diameter and lesion location. For instance, the FIRST study found that adjusting MLA values based on vessel diameter could improve the correlation between IVUS and FFR, though the correlation remained modest.^[20,21]^ Furthermore, MLA thresholds differ for different vessels, with studies suggesting distinct cutoff values depending on the coronary artery’s diameter and location.^[5]^

In our study, logistic regression analysis in the FFR group revealed a strong correlation between FFR < 0.80 and lesions located in the LAD, as well as lesion length. Some studies have shown that for patients with severely narrowed coronary arteries, there is no significant correlation between FFR values and lesion length. However, for patients with moderately narrowed coronary arteries, there is a significant negative correlation between FFR values and lesion length (r = −0.79, *P* < .0001), with a predicted lesion length for FFR < 0.8 considered >16.1 mm (sensitivity, 86%; specificity, 94%).^[22,23]^ The FIRST study also found a strong correlation between FFR < 0.8 and lesion length. This indicates that borderline lesions with a large perfused myocardial territory should be given sufficient attention, especially when the lesion length is considerable.

In our study, despite the lower occurrence of MACE in both the FFR and IVUS groups compared to the medical therapy group at the 1-year follow-up, there was no statistically significant difference. This may be attributed to the relatively small sample size and short follow-up duration. Increasing the sample size and extending the follow-up period could potentially yield more beneficial clinical outcomes.

## 5. Conclusion

Our study found that there were no significant differences in MACE between the 3 groups (IVUS, FFR, and angiography-guided PCI). These results suggest that, within the context of intermediate coronary lesions, the use of intravascular ultrasound (IVUS), FFR, or traditional angiography for guiding PCI does not result in differing outcomes in terms of MACE. Further studies with larger cohorts may be needed to confirm these findings and explore potential subgroups where specific guidance strategies could offer benefits.

## Author contributions

**Conceptualization:** Huiting Wu, Xingan Wu, Wen Yu, Baozhen Tan, Jilin Xu.

**Data curation:** Huiting Wu, Xingan Wu, Wen Yu, Baozhen Tan, Jilin Xu.

**Formal analysis:** Huiting Wu, Xingan Wu, Wen Yu, Baozhen Tan.

**Funding acquisition:** Jilin Xu.

**Investigation:** Wen Yu, Han Wang, Baozhen Tan.

**Methodology:** Wen Yu, Han Wang, Baozhen Tan.

**Supervision:** Han Wang.

**Validation:** Xingan Wu, Liang Hou.

**Visualization:** Xingan Wu, Liang Hou, Jilin Xu.

**Writing** – **original draft:** Huiting Wu, Jilin Xu.

**Writing** – **review & editing:** Huiting Wu, Jilin Xu.
